# Hidden nutritional inadequacy in U.S. children with obesity: Evidence from National Health and Nutrition Examination Survey 2021–2023

**DOI:** 10.1016/j.obpill.2026.100312

**Published:** 2026-08-01

**Authors:** John T. Stutts, Yong S. Choe, Linlin Fan

**Affiliations:** a Abbott Nutrition, 900 Easton Square Place, Columbus, OH, 43219, USA; b Pennsylvania State University, 208 Armsby Building, University Park, PA, 16802, USA

**Keywords:** Biomarkers, Childhood, Dietary intake, Obesity, Protein, Public health

## Abstract

**Background:**

Childhood is a critical period of growth and development with high nutritional demands, yet many U.S. children do not meet recommended nutrient intakes. This study assessed obesity prevalence, dietary intake, and vitamin D status among U.S. children aged 3–11 years.

**Methods:**

This brief report used cross-sectional data from the National Health and Nutrition Examination Survey (NHANES) collected between August 2021 and August 2023. Children aged 3–11 years with dietary and anthropometric data and no reported dietary supplement use were included (n = 544). Obesity was defined as body mass index (BMI) at or above the 95th percentile for age and gender. Dietary intake was assessed using first-day 24-h dietary recall data. Survey-weighted generalized linear models adjusted for age, gender, and race/ethnicity were used to compare dietary intake and vitamin D biomarkers between children with and without obesity.

**Results:**

The prevalence of obesity was 20.1% (95% confidence interval (CI): 16.8–23.4). Children with and without obesity consumed similar amounts of total energy, protein, carbohydrate, fat, and vitamin D (all *P* > 0.05). However, children with obesity had significantly (*P* ≤ 0.05) lower protein intake relative to body weight and more than fivefold higher odds of consuming protein below the Estimated Average Requirement (odds ratio (OR) 5.15, 95% CI 2.76–9.62; *P* < 0.001). Children with obesity also had significantly lower circulating vitamin D concentrations (25-hydroxyvitamin D2 + D3 <50 nmol/L) and more than threefold higher odds of low vitamin D status (OR 3.41, 95% CI 1.89–6.17; *P* < 0.001).

**Conclusion:**

Among U.S. children aged 3–11 years, obesity was associated with lower protein intake relative to body weight and poorer vitamin D biomarker status despite similar absolute dietary intake. These findings suggest that children with obesity may be at increased risk of hidden nutritional inadequacy and highlight the importance of additional nutritional monitoring and individualized care.

## Graphical abstract

**Figure:**
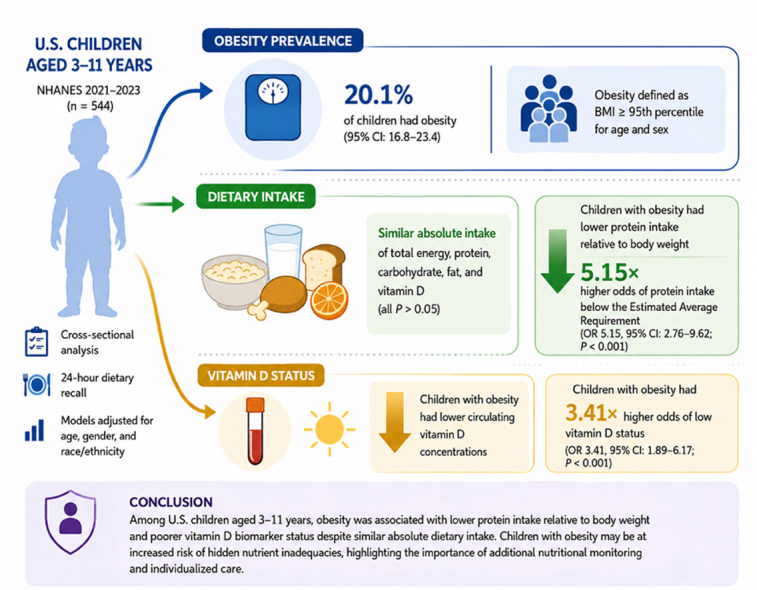

## 1 Introduction

Childhood obesity remains a major public health concern in the United States, with prevalence continuing to rise over recent decades [1]. Excess body weight during childhood is associated with a higher risk of long-term cardiometabolic conditions, including type 2 diabetes, hypertension, and cardiovascular disease [2,3]. Given that early-life health trajectories often persist into adulthood, understanding the nutritional and metabolic profiles of children with obesity is critical for informing prevention and intervention strategies.

Importantly, obesity does not necessarily indicate adequate nutrition. A growing body of evidence highlights the coexistence of excess energy intake with micronutrient deficiencies, often referred to as “hidden malnutrition” [4,5]. Children with obesity may consume diets that are high in calories but low in essential nutrients, leading to gaps in dietary quality despite sufficient or excessive energy intake [6]. This paradox underscores the need to evaluate both dietary intake and biological markers of nutritional status when studying pediatric obesity.

Protein intake is of particular importance during childhood, a period characterized by rapid growth and development. Protein requirements increase with body size, meaning that children with higher body weight may have greater absolute nutritional needs. However, prior research using National Health and Nutrition Examination Survey (NHANES) data among *adolescents* has shown that individuals with obesity are more likely to fall below the Estimated Average Requirement (EAR) for protein despite having similar absolute intake levels as those without obesity [7]. This suggests that standard dietary patterns may not adequately adjust to increased physiological demands associated with obesity.

Vitamin D is of particular interest because it plays a key role in bone development, immune function, and metabolic health, and deficiency is common among U.S. children [8]. Emerging evidence suggests that children with obesity are at higher risk of low circulating vitamin D levels, potentially due to factors such as sequestration in adipose tissue and reduced bioavailability [9]. These patterns make vitamin D an important biomarker for understanding nutritional disparities associated with obesity.

While the majority of existing pediatric studies have primarily focused on adolescents [7], less is known about dietary adequacy and nutritional biomarkers among younger children with obesity. Understanding nutritional status in younger age groups is important because early and middle childhood represent critical periods for growth, development, and long-term health trajectories. To address this gap in the literature, the present study uses NHANES data to (1) assess obesity prevalence among U.S. children aged 3–11 years, (2) compare dietary intake between children with and without obesity, and (3) evaluate differences in blood vitamin D biomarkers.

## 2 Methods

### 2.1 Study design and data source

This cross-sectional study utilized data from the National Health and Nutrition Examination Survey (NHANES) collected between August 2021 and August 2023. NHANES is a nationally representative survey of the non-institutionalized U.S. population, conducted by the National Center for Health Statistics using a complex, multistage probability sampling design. The survey integrates demographic, dietary, examination, and laboratory components, allowing for comprehensive assessment of nutritional status and health outcomes.

### 2.2 Study population

The study population included children aged 3–11 years. Participants were excluded if they had missing data on body mass index (BMI) or dietary intake. To ensure comparability in dietary intake measures, children who reported use of dietary supplements within the 30 days prior to data collection were excluded, consistent with prior NHANES-based analyses [7].

**Fig. 1:**
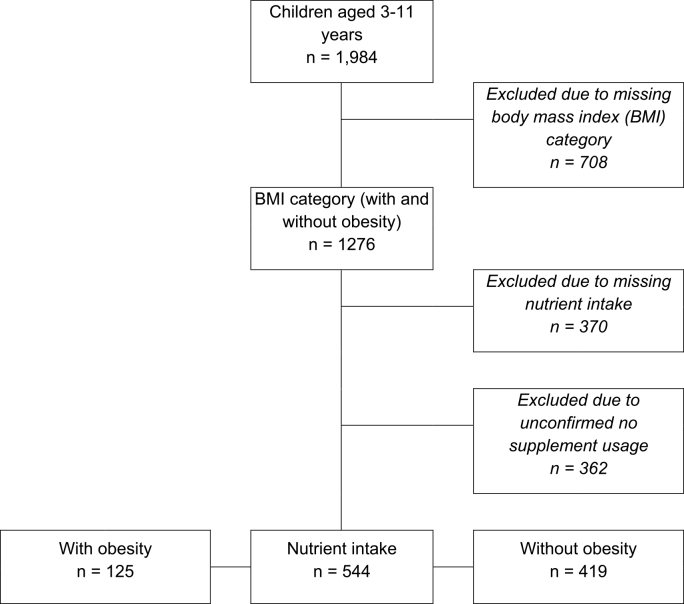
Participant Selection Flowchart National Health and Nutrition Examination Survey August 2021–August 2023 participant selection flowchart.

Participants were classified into obesity and non-obesity groups based on BMI-for-age percentiles using Centers for Disease Control and Prevention (CDC) growth charts. Obesity was defined as a BMI at or above the 95th percentile for age and sex. The final analytic sample was derived after applying all inclusion and exclusion criteria, as illustrated in Fig. 1, and included 544 children.

### 2.3 Outcome variables

The primary outcomes included the prevalence of obesity and multiple measures of dietary intake, including energy, protein, carbohydrate, and fat intake. Protein intake was assessed using first day 24-h dietary recall data and included total intake (g/day), protein-derived energy (kcal), and intake standardized by body weight (g/kg/day). We also calculated the proportion of children with protein intake below the Estimated Average Requirement (EAR) using established dietary reference standards [10]. Specifically, the EAR for protein is 0.87 g/kg/day for children aged 3 years and 0.76 g/kg/day for children aged 4–11 years. We used actual body weight to calculate the EAR, consistent with established Dietary Reference Intake guidelines [10].

Secondary outcomes focused on vitamin D status. Dietary vitamin D intake (mcg/day) was assessed using first day 24-h dietary recall data. The EAR for vitamin D intake is 10 μg/d. Blood vitamin D status was evaluated using serum 25-hydroxyvitamin D concentrations obtained from NHANES laboratory data. Low vitamin D status was defined using established clinical thresholds (25-hydroxyvitamin D2 + D3 <50 nmol/L; *Harriet Lane Handbook*, 22nd Edition) [11], and the prevalence of low vitamin D status was compared between children with and without obesity.

### 2.4 Statistical analysis

All analyses accounted for the complex survey design of NHANES using appropriate sampling weights. Following NHANES analytic guidelines, we used the survey weight for the smallest analytic subpopulation containing all variables included in each analysis. Because all dietary and vitamin D analyses included dietary intake variables, we used the Day 1 dietary sample weight, which was available for 6754 participants. Although 7626 participants had a positive phlebotomy weight for the laboratory data, the dietary weight was utilized because it represents the smaller analytic subpopulation. Dietary Day 1 sample weight was used in all analyses except for obesity prevalence, for which 2-year Mobile Examination Center (MEC) examination weight was used. Missing values were not imputed and treated as not missing completely at random for Taylor series variance estimation in SAS procedures. Continuous outcomes were analyzed using survey-weighted linear regression models, while categorical outcomes were examined using survey-weighted logistic regression. All models were adjusted for age, gender, and race/ethnicity.

Results are presented as adjusted means or proportions with corresponding standard errors or confidence intervals. Statistical significance was evaluated using two-sided tests with an alpha level of 0.05. All analyses were conducted using SAS version 9.4 (SAS Institute Inc., Cary, NC).

## 3 Results

### 3.1 Participant characteristics

Children with obesity were slightly older than those without obesity (8.0 ± 0.2 vs. 7.1 ± 0.1 years, *P* = 0.014) and were more likely to be male (52.0% vs. 44.5%). Compared with children without obesity, those with obesity had a higher proportion of Other Hispanic (25.3% vs. 13.7%) and Non-Hispanic Black (18.9% vs. 13.8%) children, and a lower proportion of Non-Hispanic White (33.0% vs. 45.4%) and Non-Hispanic Asian (4.4% vs. 6.6%) children. As expected, mean body mass index was substantially higher among children with obesity than among those without obesity (24.7 ± 0.4 vs. 16.6 ± 0.2 kg/m^2^, *P* < 0.001) (Table 1).

**Table 1:** Demographic characteristics of U.S. children aged 3–11 years with and without obesity and with nutrient intake data (NHANES August 2021–August 2023, no supplement).

Variables	With obesity (n = 125)	Without obesity (n = 419)	*P* Value
Male	63 (52.0%)	184 (44.5%)	0.255
Non-Hispanic White	36 (33.0%)	169 (45.4%)	0.065
Non-Hispanic Black	27 (18.9%)	76 (13.8%)	
Non-Hispanic Asian	6 (4.4%)	23 (6.6%)	
Mexican American	16 (13.1%)	53 (12.4%)	
Other Hispanic	31 (25.3%)	59 (13.7%)	
Other Race – Including Multi-Racial	9 (5.3%)	39 (8.1%)	
Age (years)	8.0 ± 0.2	7.1 ± 0.1	0.014§
Body Mass Index (kg/m^2^)	24.7 ± 0.4	16.6 ± 0.2	<00.001§

Continuous variables are reported as mean ± standard error, and categorical variables are reported as *n* (%). No supplement refers to participants who reported no use of dietary supplements in the past 30 days. *Abbreviations*: NHANES, National Health and Nutrition Examination Survey, n, number of participants.

### 3.2 Obesity prevalence

The prevalence of obesity among U.S. children aged 3–11 years was 20.1% (95% confidence interval (CI): 16.8–23.4).

### 3.3 Dietary intake

Table 2 presents comparisons of dietary intake between children with and without obesity. There were no statistically significant differences between groups in total energy intake, total protein intake (g/day), protein-derived energy (kcal/day), carbohydrate intake, total fat intake or vitamin D intake (all *P* > 0.05).

**Table 2:** Dietary intake and vitamin D biomarker among U.S. children aged 3–11 years (NHANES August 2021–August 2023, no supplement).

Outcome	n	With Obesity LSM±SE (n = 125)	Without Obesity LSM±SE (n = 419)	Difference LSM±SE	*P* Value
Energy (kcal)	544	1718 ± 72	1668 ± 35	49 ± 63	0.445
Protein (g)	544	58.6 ± 3.5	56.1 ± 1.5	2.4 ± 3.4	0.484
Protein (kcal)	544	234 ± 14	224 ± 6	10 ± 14	0.484
Protein per body weight (g/kg)	544	1.6 ± 0.1	2.3 ± 0.1	−0.6 ± 0.1	<00.001§
Carbohydrate (g)	544	218 ± 10	221 ± 7	−3±9	0.715
Carbohydrate (kcal)	544	871 ± 40	885 ± 26	−14 ± 36	0.715
Total fat (g)	544	69.1 ± 3.4	63.3 ± 1.0	5.8 ± 3.4	0.115
Total fat (kcal)	544	622 ± 31	570 ± 9	52 ± 31	0.115
Vitamin D (D2 + D3) (mcg)	544	5.4 ± 0.7	4.5 ± 0.3	0.9 ± 0.8	0.309
25OHD2+25OHD3 (nmol/L)	316	58.6 ± 1.7	62.8 ± 1.2	−4.2 ± 1.5	0.013§

Regression analysis adjusted for age, gender, and race/ethnicity. Obesity is defined as a body mass index (BMI) at or above the 95th percentile based on CDC sex-specific BMI-for-age growth charts. No supplement refers to participants who reported no use of dietary supplements in the past 30 days. *Abbreviations*: NHANES, National Health and Nutrition Examination Survey; n, number of participants; LSM, least squares mean; SE, standard error; §, statistically significant.

However, when protein intake was standardized by body weight, a significant difference emerged. Children with obesity had significantly lower protein intake per kilogram of body weight compared with children without obesity (*P* < 0.05), indicating that their intake may not meet higher physiological requirements.

### 3.4 Protein intake below EAR

As shown in Table 3, children with obesity had significantly greater odds of consuming protein below the Estimated Average Requirement (EAR) compared with children without obesity (odds ratio (OR) 5.15, 95% CI 2.76–9.62, *P* < 0.001). In contrast, protein inadequacy was not significantly associated with sex (*P* = 0.228) or race/ethnicity (*P* = 0.698).

**Table 3:** Prevalence of dietary intake below estimated average requirement (EAR) and low vitamin D status among U.S. children aged 3–11 years (NHANES August 2021–August 2023, no supplement).

Outcome	n	Odds Ratio, 95% CI	*P* Value
Below EAR for protein	544	5.15, 2.76–9.62	<00.001§
Below EAR for vitamin D	544	1.06, 0.29–3.93	0.924
Low vitamin D (25OHD2+25OHD3 <50 nmol/L)	316	3.41, 1.89–6.17	<00.001§

Logistic regression analysis adjusted for age, gender, and race/ethnicity. Obesity is defined as a body mass index (BMI) at or above the 95th percentile based on CDC sex-specific BMI-for-age growth charts. No supplement refers to participants who reported no use of dietary supplements in the past 30 days. *Abbreviation*s: NHANES, National Health and Nutrition Examination Survey; n, number of participants; CI, confidence interval; §, statistically significant.

### 3.5 Vitamin D findings

Although vitamin D intake did not differ significantly between children with and without obesity, differences emerged in the blood biomarker. Children with obesity had significantly lower circulating vitamin D concentrations compared with those without obesity (25OHD2+25OHD3: 58.6 ± 1.7 vs. 62.8 ± 1.2 nmol/L; difference: −4.2 ± 1.5; *P* = 0.013; n = 316). In addition, children with obesity had significantly higher odds of low vitamin D status based on the biomarker measure (OR 3.41, 95% CI 1.89–6.17; *P* < 0.001; n = 316). The odds of low vitamin D status were also higher among females (OR = 0.80, 95% CI: 0.33–1.95) and racial/ethnic minority groups compared with their counterparts. Relative to non-Hispanic White children, the odds of low vitamin D status were substantially higher among non-Hispanic Black children (OR = 41.59, 95% CI: 14.00–123.61), non-Hispanic Asian children (OR = 29.90, 95% CI: 6.29–142.15), children of other or multiracial backgrounds (OR = 7.32, 95% CI: 2.49–21.52), Mexican American children (OR = 5.93, 95% CI: 2.00–17.58), and other Hispanic children (OR = 5.84, 95% CI: 2.44–13.97).

## 4 Discussion

This study examined obesity prevalence, dietary intake, and vitamin D status among U.S. children aged 3–11 years using nationally representative NHANES data. We found that 20.1% of children had obesity. Although children with and without obesity consumed similar amounts of total energy, protein, carbohydrate, and fat, children with obesity had significantly lower protein intake relative to body weight and more than fivefold higher odds of consuming protein below the Estimated Average Requirement compared to those without obesity (OR 5.15, 95% CI 2.76–9.62). Children with obesity also had significantly lower circulating vitamin D concentrations and more than threefold higher odds of low vitamin D status (OR 3.41, 95% CI 1.89–6.17). Together, these findings suggest that excess body weight can coexist with relative nutritional inadequacy, highlighting an underrecognized form of hidden malnutrition in pediatric obesity [12,13].

The central contribution of this study is the distinction between absolute nutrient intake and physiologic adequacy. Research on pediatric obesity often focuses on excess caloric intake, implicitly assuming that greater food consumption reflects sufficient nutrient intake. Our findings challenge this assumption. Because protein requirements scale with body weight, children with larger body size have greater physiologic protein needs [14]. In this context, similar absolute protein intake across weight groups does not imply equivalent nutritional adequacy. Children with obesity consumed comparable amounts of protein overall, yet their intake relative to body size was significantly lower, leading to markedly greater odds of intake below the EAR. These findings suggest that nutrient adequacy should be evaluated relative to physiologic demand rather than total intake alone.

These findings have important implications for clinical care and nutritional surveillance. Pediatric obesity management often prioritizes calorie reduction and weight control, while nutrient adequacy receives less attention. However, energy-dense, nutrient-poor diets may allow children to exceed caloric needs while still failing to meet essential nutrient requirements [4,6]. Adequate protein intake during childhood is particularly important for growth, lean mass development, metabolic regulation, and satiety [15]. Insufficient protein intake relative to body size may therefore have implications for body composition and long-term metabolic health during critical periods of growth and development. Our findings support a broader shift from viewing obesity solely as excess adiposity toward recognizing the coexistence of overnutrition and undernutrition within the same child.

We also found significantly lower circulating vitamin D concentrations and substantially greater odds of low vitamin D status among children with obesity despite similar dietary vitamin D intake. This divergence between intake and biomarker status suggests that obesity may alter nutrient metabolism and utilization. Proposed mechanisms include sequestration of vitamin D in adipose tissue, altered endocrine regulation, reduced bioavailability, and volumetric dilution effects associated with larger body size [[16], [17], [18]]. These findings further suggest that dietary intake measures alone may underestimate nutritional vulnerability in children with obesity.

More broadly, our findings reinforce the need to move beyond calorie-focused approaches to childhood obesity. Malnutrition and obesity are often treated as separate conditions; however, our results support growing evidence that they may coexist within the same population and even within the same individual [6,13]. Focusing exclusively on calories or body weight may therefore overlook important dimensions of nutritional risk. Among children participating in weight reduction programs, these nutritional gaps may be further exacerbated without appropriate nutritional support and intervention. Early strategies that improve diet quality, nutrient density, and nutritional adequacy—not simply caloric balance—may be important for supporting healthier growth trajectories and long-term metabolic health in children with obesity. These findings underscore the importance of encouraging balanced dietary patterns that provide adequate essential nutrients while supporting healthy weight management. Future intervention studies are needed to determine the most effective approaches for improving nutritional adequacy in this population.

## 5 Limitations

This study has several limitations. First, the retrospective design of NHANES precludes causal inference between obesity and nutritional inadequacy. For example, serum vitamin D concentrations are influenced by factors beyond dietary intake, including season, geographic location, sunlight exposure, physical activity, skin pigmentation, consumption of vitamin D–fortified foods, and socioeconomic status. Because not all of these factors were available or included in our analyses, residual confounding may have influenced the observed associations. Second, dietary intake was self-reported and may be subject to recall bias and reporting errors. A single 24-h recall may not reflect an individual's usual nutrient intake and could result in misclassification of whether a child meets the EAR, potentially attenuating the observed associations. Furthermore, dietary intake for younger children was reported by a parent or caregiver rather than by the child directly. As a result, the dietary recalls may be subject to proxy-reporting error, particularly for foods consumed outside the home, although this limitation is common in pediatric dietary surveys. Third, while children taking dietary supplements were excluded to ensure a more homogeneous comparison of nutrient intake from foods alone, excluding these children may limit the generalizability of our findings to the broader pediatric population. Fourth, children with overweight were classified together with those without obesity, which may have masked differences in dietary intake and nutritional status across weight categories. Another limitation is the relatively small sample of children with obesity compared with children without obesity. The imbalance in sample sizes may have reduced statistical power to detect differences between groups, and larger studies are needed to confirm our findings. Finally, our analytic sample was restricted to children with complete data on BMI category, dietary intake, and confirmed non-use of dietary supplements. Because of missing data on BMI category and dietary intake, we cannot determine whether systematic differences existed between included and excluded participants. Consequently, our findings may not be fully generalizable to all U.S. children aged 3–11 years, particularly if excluded children differed in nutritional characteristics.

## 6 Conclusion

Among U.S. children aged 3–11 years, 20.1% met criteria for obesity. Although children with obesity consumed similar absolute amounts of protein as those without obesity, they had significantly lower protein intake relative to body weight and more than fivefold higher odds of consuming protein below the Estimated Average Requirement. Children with obesity also had significantly lower circulating vitamin D concentrations despite similar dietary vitamin D intake. These findings suggest that children with obesity may be at increased risk of hidden nutritional inadequacy and highlight the importance of evaluating nutrient adequacy relative to physiologic requirements rather than total intake alone. Future longitudinal studies should examine how nutrient inadequacy in childhood obesity affects growth, body composition, and long-term metabolic health outcomes.

### 6.1 Key takeaway clinical messages

• Children with obesity may consume similar absolute amounts of protein as their peers while still failing to meet protein requirements relative to body size.
• Obesity does not necessarily indicate adequate nutritional status, and children with obesity may be at increased risk of hidden nutrient inadequacies.
• Pediatric obesity management should incorporate assessment of diet quality and nutrient adequacy, including additional nutritional monitoring and individualized care.

## CRediT author statement

• Conceptualization, J.S., C.Y.; Methodology, C.Y.; Software, C.Y.; Formal Analysis, C.Y.; Resources, J.S.; Data Curation, C.Y.; Writing - Original Draft, L.F.; Writing - Review & Editing, L.F.; Supervision, J.S.; Project Administration, L.F.

## Declaration of generative AI and AI-assisted technologies in the writing process

During the preparation of this work the authors used OpenAI's tool ChatGPT to refine and proofread the manuscript. After using this tool, the authors reviewed and edited the content as needed and take full responsibility for the content of the publication.

## Source of funding

This manuscript was not funded by any specific grant from funding agencies in the public, commercial, or not-for-profit sectors. As employees of Abbott Laboratories, J.S. and C.Y. receive salaries for their professional responsibilities.

## Declaration of competing interests

J.S. and C.Y. are employees and stockholders of Abbott (Abbott Park, IL, USA).
